# Clinical Holistic Medicine: Chronic Infections and Autoimmune Diseases

**DOI:** 10.1100/tsw.2005.23

**Published:** 2005-02-23

**Authors:** Søren Ventegodt, Joav Merrick

**Affiliations:** ^1^ The Quality of Life Research Center, Teglgårdstræde 4-8, DK-1452 Copenhagen K, Denmark and The Scandinavian Foundation for Holistic Medicine, Sandvika, Norway; ^2^ National Institute of Child Health and Human Development, Office of the Medical Director, Division for Mental Retardation, Ministry of Social Affairs, Jerusalem and Zusman Child Development Center, Divisions of Pediatrics and Community Health, Ben Gurion University, Beer-Sheva, Israel

**Keywords:** quality of life, QOL, philosophy, human development, holistic medicine, public health, holistic health, holistic process theory, life mission theory, infectious diseases, autoimmune diseases, consciousness-based medicine, Denmark

## Abstract

The consciousness-based (holistic) medical toolbox might be useful in general practice and in cases of recurrent infections and chronic infection or inflammation. From our clinical experiences, there is hope for improvement from a number of diseases caused by disorders affecting the regulation of the immune system when the physician includes the holistic medical approach.Our scientific understanding of the connection between consciousness and cellular order is still limited. Consciousness-based holistic medicine removes (as explained by the holistic process theory of healing) the “blockages” in the tissues of the body and facilitates function and informational exchange of the cells of the body. Many blockages and repressed feelings in an area would imply “noise and disturbances” on the level of intercellular communications, which in turn means major difficulties for the cells of the immune system. For this they are totally dependent on the body information system, which the holistic treatment aims to recover. Processing the blockages increases the coherence of the cells and organism, thus increasing the intercellular flow of information in the area and thus strengthening the immune defense and healing the disease. The area of clinical holistic medicine is going through a rapid development and the toolbox of consciousness-based medicine is available for dealing with many diseases arising from disturbances in the regulation of the immune system. Holistic medicine has yet to be better explained scientifically and our proposed holistic cures have yet to be documented clinically. We invite the medical community to cooperate on this important challenge.

## INTRODUCTION

Autoimmune diseases and chronic infections are mostly resistant to biomedical treatments and most therapy is mainly palliative and substitution. For many years, the spontaneous remission of autoimmune disease (like the spontaneous remissions of chronic infections) has been puzzling to physicians, with the spontaneous remission of type I diabetes being the most intensively studied[1,2,3,4,5,6]. The observed dynamics strongly indicate that there are factors involved that we do not understand or control.

The holistic approach might be relevant in general practice when our biomedical tools prove inadequate, for instance, against recurrent infections in the throat, lungs, or abdomen in autoimmune diseases such as type 1 diabetes, sclerosis, and rheumatoid arthritis and in chronic infections such as hepatitis, mononucleosis, and possibly also in fighting the HIV infection.

If not properly regulated, the immune system can cause a variety of diseases. It comprises billions of cells distributed throughout the body and to function well, it is highly dependent on intercellular communication. The immune system receives a lot of its information from the cells and tissues that it aims to protect. The cells in an area (for instance, connective tissue cells in an infected finger) tell the immune system about an impending danger that calls for immune defense. We know this from immunization studies where we often have to give a primer that is an irritant to tissues to raise an immune defense against the injected antigen[7,8].

As our scientific understanding of consciousness is very limited, it is difficult to give a precise mechanical explanation as to how holistic medicine works. As an introduction, we understand that consciousness-based holistic medicine removes “blockages” (in a fairly complex, but understandable way, explained by the holistic process theory, see below) that disturb the tissues and cells of the body while processing the somatized feelings and gestalts placed in the tissue of the relevant organ(s) at the moment of shock and trauma; this approach seems appropriate for strengthening the immune defense. The explanation is that the wholeness of the person represses an unbearable feeling to a part of the body in order for this part to hold it until the person can take care of it emotionally. It is a sheer survival mechanism that works by allocating the tissue from its physiological purpose to the purpose of holding the gestalt. This disturbs the cells, as they cannot process the job at the present time, so they become less an integral part of the body than before. Thus, holistic healing is needed to make the patient whole again. After a short review of our work on holistic healing, we shall explain more about our understanding of the theoretically difficult psychobiological connection.

## CLINICAL HOLISTIC MEDICINE

The life mission theory[9,10,11,12,13,14] states that everybody has a purpose of life or huge talent. Happiness comes from living this purpose and succeeding in expressing your core talent. To do this, it is important to develop as a person into what is known as the natural condition, a condition where you know yourself and use all efforts to achieve what is most important for you. The holistic process theory of healing[15,16,17,18] and the related quality of life theories[19,20,21] state that the return to the natural state of being is possible whenever the person gets the resources needed for existential healing. The resources needed are holding in the dimensions of awareness, respect, care, acknowledgment, and acceptance with support and processing in the dimensions of feeling, understanding, and letting go of negative attitudes and beliefs. The preconditions for holistic healing are trust and the intention for the healing to take place. Existential healing is not a local healing of any tissue, but a healing of the wholeness of the person, making him much more resourceful, loving, and knowledgeable of himself, his needs, and his wishes. In letting go of negative attitudes and beliefs, the person returns to a more responsible existential position with an improved quality of life. The philosophical change of the person healing is often a change towards preferring difficult problems and challenges, instead of avoiding difficulties in life[22,23,24,25,26,27,28,29]. The person who becomes happier and more resourceful often also becomes more healthy, more talented, and more able to function[30,31,32].

## THE CONTROL OF THE IMMUNE SYSTEM

How is the immune system controlled? This question is quite central if we want to raise resistance to bacteria and viruses and to help the immune system to fight an infection, or if we need to weaken the destructive attacks of the immune system against the body's own cells, which is what happens in an autoimmune disease. In type 1 (juvenile) diabetes, the attack is aimed at the insulin-producing beta cells in the islets of Langerhans. In rheumatoid arthritis, the targets are the joint capsules. In sclerosis, the attack affects more-or-less well-defined areas of the brain and its nerves.

Several theories exist on the regulation of the immune system. Biochemical theories point to chemical networks, while holistic theories center on biological information, feelings, and consciousness. Accordingly, holistic medicine explains immune system disorders in the same way as it explains imbalances in other body tissues and organs: something prevents the immune cells from doing their job at their location. Something affects the cells of the immune system, keeping necessary information from them and preventing their prompt and efficient reaction to the intrusion of an enemy. Instead, their reaction is too weak or they attack healthy tissues that should certainly not have been destroyed.

The immune system has several lines of defense. The first is mechanical: the skin and mucous membranes prevent bacteria and other microorganisms from entering our sugar-containing body fluids in order to multiply. The next barrier is a nonspecific immune response where several cells, such as the macrophages, specialize in distinguishing between the body's own cells and structures (self) and foreign cells and structures that should be destroyed (nonself). The ability to distinguish between self and nonself is the backbone of the immune system, but there is little scientific knowledge about this ability.

Interesting studies of the evolution of the immune system showed that this ability dates back to the very first living creatures on Earth. Since even the most primitive sponges, the first multicellular organisms were able to distinguish between self and nonself at the cellular level. If nonspecific immune cells fail to destroy an invading enemy, the specific immune system springs into action. It works by means of antibodies, especially against bacteria and killer cells, especially against viruses, which kill all virus-infected cells before they can pass on the virus.

According to the holistic medicine theory, the way that the specific and nonspecific immune systems work depends on the number of blockages in the body. Many blockages and repressed feelings in an area would imply a lot of noise and disturbances in intercellular communications, which in turn means major difficulties for the cells of the immune system when they need to distinguish between self and nonself. For this they are totally dependent on the body's information system.

## FINE TUNING THE IMMUNE SYSTEM

If the patient is free from the disturbing blockages in the body, he or she will experience improved quality of life and become a more coherent person. The information systems of the body will again be able to deliver precise and correct information to the immune cells. In this way, the immune system is fine tuned and better at eliminating foreign organisms, while letting the cells of the body live.

At this moment, we do not know how effective holistic medicine is against, for instance, HIV infection. Nor do we know how irreparable the damage caused by autoimmune diseases like type 1 diabetes and sclerosis. It is hoped that there are viable stem cells in the pancreas that can begin to produce insulin once the immune system stops destroying all the insulin-producing cells. As for sclerosis, the brain is known to have great potential, so if the immune system ceases to make plaque, or holes in the brain, even very poorly sclerotic patients should be able to recover dramatically once the autoimmune attacks cease.

## CASE PRESENTATIONS FROM THE HOLISTIC RESEARCH CLINIC

Clinical holistic health research work is an emerging field with small numbers of patients that we try to learn from, but judging from the clinical experiences, there is hope of improvement and recovery from a large number of diseases that have been caused by disorders affecting the regulation of the immune system.

> **Male, aged 47 years, with chronic sore throat and diabetes:** First visit: Chronic sore throat for months, sensation of a "lump" in his throat. On examination: Strep A: negative. Oral cavity: No redness or coating, swelling around the larynx. /Chronic irritation of larynx/History of /NIDDM/. He is finding his work as an auditor difficult at the moment and would like to lose weight. This might enable him to go back to the tablets instead of injections of insulin. Weighs 118 kg. Wife says that he snores. His father has just died. We talk about only eating to satisfy his hunger. EXERCISE: Avoid forcing your voice when you are speaking/working.
>
> Second visit: Throat complaints resolved since last visit due to the exercise. His throat complaint seems to be caused by his feeling that he cannot get his messages across. This makes him check his own expressions, so that tension builds up in his throat. He acknowledges this correlation and performs the exercise of trying to avoid the feeling of putting pressure on himself. That solves the matter and rids him of a month-long chronic throat condition.
>
> **Female, aged 38 years with chronic cough:** Bronchial nonproductive cough on and off for years. Taking strong Pectyl [combination drug with opioid] with inadequate effect. On examination: Auscultation of the lungs: normal. Severe tenderness on sternum, cervical muscles very tense, trigger points positive on upper and lower limbs. Obviously something is not quite right. The patient should find out herself what is wrong. Follow up.

This patient was coughing and a strong cough mixture with opioid was not working. The trigger points in the chest region are very tender. Trigger points are the sites in the body where the physician can best reveal muscle pain and tenderness by touch and they coincide largely with the Chinese points of acupuncture. What is it about? The physician may have a strong suspicion, but that will not help the patient who must look into the matter herself. We hope that she will return and relate all her difficulties in liking herself and enjoying her life, however, it is undeniably simpler to take cough medicine with opioid than to think about blockages in the chest region. Unfortunately the easy solution did not help. That leaves the difficult one. Although holistic physicians often have a very precise idea of what is wrong, we cannot tell the patients; they must get in touch with their innermost feelings to understand what is happening in their lives. At that point, our words would only cause disturbance and blockage. So it is not that we are unwilling to help when we hold back and laconically write in the case record that "the patient should find out for herself what is wrong". In this respect, consciousness-based medicine, which is about developing the patient's consciousness and understanding, differs substantially from biomedicine, where the knowledge of the physician can be communicated verbally to the patient without any major difficulties.

> **Female, aged 46 years, with migrating physical irritation following daughter's suicide:** First visit: Arrive four days after onset of tonsillitis. Last night, she was swelling like a toad and she presents with massive oedema on the face, chest and forearms. She is barely able to look out of her eyes and feels a burning sensation in the face. On examination: No fever. No redness or coating on the tonsils. Strep A test negative. No

> redness, but pronounced swelling and heat in the regions mentioned. /Hypersensitivity reaction following tonsillitis/ Prescribe antihistamine Zyrtec [cetirzine] 10 mg as required.
>
> Second visit: Problem 1: Good effect of Zyrtec on facial oedema, now fine. Problem 2: Three weeks of coughing with clear phlegm, “migrating irritation”. Auscultation of the lungs: normal. Prescribe Pectyl [combination drug with opioid] cough mixture. Problem 3: Her daughter committed suicide three years ago and she has not recovered yet. Wants an appointment for conversation.

The problems are closely interconnected. She has not processed her daughter’s suicide properly. Painful feelings of guilt and sorrow are left in her system and cause disturbances that migrate within her system, as is the way with such blockages. When irritation is seen to migrate from place to place and from organ to organ, to us that is a sure sign of a psychosomatic complaint. Often, there is an ongoing emotional issue that can be resolved. Afterwards it will all fall into place and sometimes it is possible to resolve ten health issues in one trial when we get to the root of the problem.

> Third visit: We talk about the daughter’s suicide, which took place right after her separation. She left three young children and the patient fails to understand this. We talk about having a broken heart, the greatest pain in life. The patient goes over the event, sobbing. EXERCISE for next time, which the patient believes she can handle: Write down what happened, what did you feel. Write for 10 minutes a day and put the writing paper away in an envelope till next day.

Actually, it is more than just a trauma when your daughter commits suicide. It is a personal disaster, where no mother can avoid feeling guilt because you wonder if you have really been a good enough mother. All children end up feeling let down by their mother (and/or father), so there is plenty to tackle. If the mother goes through with the processing, which personal disasters like that require, she will surprisingly gain a new life from her daughter’s death. Native Americans offer the words of wisdom that *the life she has given will return to her*. Note that the envelope will guard the hot topic until next day, so that it does not bother the patient in the meantime. After all, you cannot eat an elephant at one gulp, so where to put it between meals?

> **Male, aged 55, years with severe rheumatoid arthritis in knees:** Tenderness in both knees. On examination: Left knee tenderness on medial side and in superior muscles. Joint line free, no accumulations, no drawer signs or other symptoms. Left knee: Patellar crepitation, no other pathological finding in spite of serious radiographic diagnosis of severe arthrosis. Clinically the patient’s knee is fine today apart from the pain. EXERCISE: Massage around the knees; raised awareness of knee movements, when walking, for instance 30-minute walks twice a week with a comfortable gait.

The situation seems hopeless, but it is not. Even very severe rheumatoid arthritis may appear without severe pain, or there may be the most excruciating pain without any evidence of arthritis or articular damage. We believe that pain and blockage are closely related and once the blockage is lifted, the pain will disappear. Since the blockage also caused articular and cartilage degeneration, the knee will be free from pain (including the way we see it) and begin to heal. Sometimes, cartilage becomes as good as new on the X-rays, so we do not regard an X-ray as a final verdict, but as a report on the current condition. Unless there is evidence to the contrary, we expect our arthritic patients to become well again. Why would they not, if the body is nothing but a colony of cells controlled by information flows that we can optimize, fine tune, and adjust?

**Female, aged 35 years, with type 1 diabetes:** The patient wants to be cured of her type 1 diabetes that has lasted 19 years. The project is uncertain, but perhaps the pancreatic stem cells can propagate to form new insulin-producing beta cells once the autoimmune response cease as a result of the processing of gestalts in the pancreas. BP 130/75. Occasional eye problems, but not today. Some white spots in one eye. The picture varies a little. Other problems: 1. Professionally a perfectionist. 2. “I am happy – I choose to be,” the patient says. On a scale from 1 to 10, how happy? 10! I have the best friends and the best family in the world. She no longer has a boyfriend. “I can handle the sexual drive” she says. She sounds very convincing, when stating that she can suppress it. She is very much afraid of becoming unhappy, when the facade cracks. 3. Worries too much about certain people and certain things – very worried about her family and her three brothers. Mother died of cancer two years ago. She cries when talking about her. “I will not accept that life hurts. I cannot do without them. I totally rely on them.” 4. She cannot bear to fall out with someone. “Afraid of conflicts?” I suggest. But no, conflicts are fine; they should merely be turned into positive experiences. EXERCISE: “Mentally I am not very strong,” she says – write two A4 pages about that for next time. Describe your mental frailty. I am rational – not emotional.

Why should it not be possible to make the stem cells mature into insulin-producing cells so that they can cure diabetes? In theory, this should be possible when the imbalances behind the diabetes have been removed with the patient’s mind set on healing. This project looked very optimistic, but the patient lost some of her enthusiasm when she realized that it will take years of hard work on the odd chance of achieving a doubtful result.

## SCLEROSIS

The following case history concerns a patient with sclerosis. Sclerosis is an autoimmune disease where the patient’s own immune defense attacks areas in the brain. Sclerosis generally causes disability and it is one of the diseases that we cannot do much about with established biomedicine. Here, I (SV) am trying to help a young man to improve his quality and meaning of life by mobilization of his inner resources based on the life mission theory[2].

**Male, aged 32 years, with sclerosis:** First quality-of-life (QOL) conversation: A period of fatigue and attacks, paraclinical findings with changes on the MRI scan and slightly elevated IgG index plus evoked potentials showing effects corresponding to visual evoked potential on the right eye, which leads us to the conclusion that here we have a demyelisation disorder. /Multiple sclerosis/ Several months ago, the patient tried to commit suicide and now wishes to have a conversation.

Second QOL conversation: Conversation about sclerosis (MS). I explain the disease: autoimmunity. During the last four months, the patient has also suffered from a buzzing in right ear. The patient went to an ear specialist, who thinks that the hearing loss is due to MS. He also has problems with right eye on exertion. On examination: Muscles soft, weak, body emaciated; he has lost 12 kg over the last couple of months. Something seems wrong with his will to live. He has always felt that he would not grow very old. EXERCISE: I recommend that he writes his autobiography, looking for problematic situations, especially where he opts out of life. What happened? What did he decide? Can return in six weeks for another conversation.

Writing an autobiography is a highly effective tool in order to make people realize what is wrong with them. The exercise should be given at the right time, otherwise it will not work and it cannot be applied again later. Timing is everything according to the purpose of life theory because the patient must have resources and motivation for the assignments given. Otherwise, the patient becomes overwhelmed and simply does not return, or at least not with a will to cooperate. Here, the exercise solved the ear problems.

> Third QOL conversation: Ear problems now gone. He brings a 23-page story of his life. Good work and he is encouraged to continue, focusing on emotional events that he should describe in detail. It is important that he regards this work as strictly confidential. The patient should do an EXERCISE accounting for his life energy (what provides and what requires energy) and his enjoyment of life (what provides and what requires), and for next time he should look at how to optimise his life energy and enjoyment of life.

> Fourth QOL conversation: The patient is feeling well, no major ear or eye conditions or any other sclerotic symptoms. He has migraine again, which he has not experienced since he developed sclerosis. I suggest that this could be a step forward, since he is again able to feel something in his head. He has not been able to feel anything during the episode of sclerosis. He has done his homework – good lists. I (SV) particularly noticed: what provides life energy: “When I feel well and have done something that I am proud of.” The patient has also discovered the statement “What’s the point.” This is an interesting statement, which to him means something like: “My life is a waste of time.” Talk about the meaning of life. EXERCISE: Write 1000 times: “What’s the point?” and see what feelings it provokes in you.

A return to previous stages of the disease and the recurrence of minor complaints that preceded the current major ones are things that we would usually regard as healthy signs — signs that things are moving in the right direction and that the patient is slowly, but steadily, working his way backwards and down through his personal “rubbish heap” towards the gold representing the purpose of life.

> Fifth QOL conversation: The patient has done his homework and written 300 to 400 times the statement: “What is it all about … that we are born and die,” the patient says. When his mother died when he was nine, he repressed all his feelings and made the decision that life was close to meaningless – expressed by the statement “What’s the point.” He let go of this statement. No feelings emerge except meaninglessness, so I suspect that he still restrain his feelings. The way I see it the patient is making good progress. He should continue working on it by himself.

We find that this patient’s course of treatment is one of those that brings us real satisfaction. His recovery was fast and the development project surpassed our expectations. The patient was cooperative, committed, and diligent; he accepted even very hard exercises, which means good and rapid progress. Already after five sessions, he can work independently with things. The symptoms went and we expect that he will do well in spite of a very serious diagnosis. How he will actually do will become apparent over the next 10 years.

The way we see it, autoimmune diseases are caused by blockages that disrupt the ability of the immune system to distinguish between self (the body’s own tissues) and nonself (foreign cells and elements). When blockages are processed, the immune defense re-establishes the ability to distinguish between self and nonself, and the autoimmune attacks will cease. Let it be said that there are many layers in this patient’s consciousness that have not been processed, so normally new episodes of sclerosis could emerge, which would then require new therapeutic sessions. Only when the patient’s life is coherent on the emotional level will he stay healthy without the risk of a relapse.

## CONCLUSION

Our scientific understanding of the connection between consciousness and cellular order is very limited and it is difficult to give a precise mechanical explanation for the healing mechanisms of holistic medicine on a biological level. Consciousness-based holistic medicine removes “blockages” that disturb the tissues and cells of the body. Holistic medical theory attempts to explain how the disturbances of the information to the cells influence and weaken both the specific and nonspecific immune systems. Many blockages and repressed feelings in an area would imply disturbances in intercellular communications, which in turn means major difficulties for the cells of the immune system when they need to distinguish between self and nonself.

The field of clinical holistic medicine is going through a rapid development and the toolbox of consciousness-based medicine now seems adequate for dealing with many diseases arising from disturbances in the regulation of the immune system. The proposed holistic cures have yet to be documented clinically. We invite all members of the international medical community to participate in the important project of developing consciousness-based medicine. For the purpose of developing an evidence-based holistic medicine, we have developed an affordable documentation method especially useful for documenting the effect of alternative complementary and holistic medicine[15] and we hope that this affordable and simple method will be taken widely into use.

As our final remark, we strongly suggest that research in holistic medicine, with its promising new cures for a long series of diseases, receives more financial support from governments and foundations, to further accelerate the development of a new medicine that is not dependent on expensive drugs and can be of use for poor people and even in the poor regions of the world.

## ACKNOWLEDGMENTS

This study was supported by grants from IMK Almene Fond. The quality of life research was approved by the Copenhagen Scientific Ethical Committee under number (KF)V.100.2123/91.

## BIOSKETCHES

**Søren Ventegodt, MD,** is the director of the Quality of Life Research Center in Copenhagen, Denmark. He is also responsible for a Research Clinic for Holistic Medicine in Copenhagen and is a popular speaker throughout Scandinavia. He has published numerous scientific or popular articles and a number of books on holistic medicine, quality of life, and quality of working life. His most important scientific contributions are the comprehensive SEQOL questionnaire, the very short QoL5 questionnaire, the integrated QOL theory, the holistic process theory, the life mission theory, and the ongoing Danish Quality of Life Research Survey, 1991–94 in cooperation with the University Hospital of Copenhagen and the late professor of pediatrics, Bengt Zachau-Christiansen, MD, PhD. E-mail: ventegodt@livskvalitet.org. Website: http://www.livskvalitet.org

**Joav Merrick, MD, DMSc,** is professor of child health and human development affiliated with the Zusman Child Development Center, Division of Pediatrics and Community Health at the Ben Gurion University, Beer-Sheva, Israel; the medical director of the Division for Mental Retardation, Ministry of Social Affairs, Jerusalem; and founder and director of the National Institute of Child Health and Human Development. He has numerous publications in the field of child health and human development, rehabilitation, intellectual disability, disability, health, welfare, abuse, advocacy, quality of life and prevention. Dr. Merrick received the Peter Sabroe Child Award for outstanding work on behalf of Danish Children in 1985 and the International LEGO-Prize (“The Children’s Nobel Prize”) for an extraordinary contribution towards improvement in child welfare and well being in 1987. E-mail: jmerrick@internet-zahav.net. Website: www.nichd-israel.com
